# 2-(4-Eth­oxy­carbon­yl-5-methyl-1*H*-1,2,3-triazol-1-yl)acetic acid mono­hydrate

**DOI:** 10.1107/S1600536810043813

**Published:** 2010-10-31

**Authors:** Gai-Gai Wang, Hong Zhao

**Affiliations:** a School of Chemistry and Chemical Engineering, Southeast University, Nanjing 210096, People’s Republic of China

## Abstract

The title compound, C_8_H_11_N_3_O_4_·H_2_O, was synthesized by reaction of 2-azido­acetic acid and ethyl acetyl­acetate. In the crystal packing, mol­ecules are linked by strong inter­molecular O—H⋯N and O—H⋯O hydrogen bonds into double layers parallel to the *ab* plane.

## Related literature

For the biological activities of triazole derivatives, see: Olesen *et al.* (2003 ▶); Tian *et al.* (2005 ▶). For the synthesis, see: El Khadem *et al.* (1968 ▶). For related structures, see: Lin *et al.* (2008 ▶); Xiao *et al.* (2008 ▶); Zhao (2009 ▶).
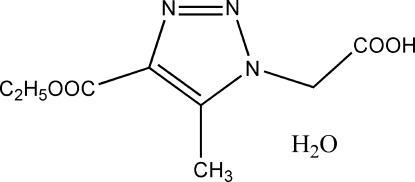


## Experimental

### 

#### Crystal data

C_8_H_11_N_3_O_4_·H_2_O
*M* *_r_* = 231.21
Monoclinic, 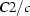

*a* = 18.6082 (15) Å
*b* = 8.2295 (15) Å
*c* = 14.986 (2) Å
β = 92.050 (5)°
*V* = 2293.4 (6) Å^3^
*Z* = 8
Mo *K*α radiation
μ = 0.11 mm^−1^
*T* = 295 K
0.35 × 0.32 × 0.28 mm

#### Data collection

Rigaku SCXmini diffractometer
Absorption correction: multi-scan (*CrystalClear*; Rigaku, 2005 ▶) *T*_min_ = 0.960, *T*_max_ = 0.970
10958 measured reflections
2490 independent reflections
1536 reflections with *I* > 2σ(*I*)
*R*_int_ = 0.051

#### Refinement

*R*[*F* ^2^ > 2σ(*F* ^2^)] = 0.066
*wR*(*F* ^2^) = 0.189
*S* = 1.09
2490 reflections
148 parameters
H-atom parameters constrained
Δρ_max_ = 0.28 e Å^−3^
Δρ_min_ = −0.20 e Å^−3^

### 

Data collection: *CrystalClear* (Rigaku, 2005 ▶); cell refinement: *CrystalClear*; data reduction: *CrystalClear*; program(s) used to solve structure: *SHELXS97* (Sheldrick, 2008 ▶); program(s) used to refine structure: *SHELXL97* (Sheldrick, 2008 ▶); molecular graphics: *SHELXTL/PC* (Sheldrick, 2008 ▶); software used to prepare material for publication: *SHELXTL/PC*.

## Supplementary Material

Crystal structure: contains datablocks I, global. DOI: 10.1107/S1600536810043813/rz2507sup1.cif

Structure factors: contains datablocks I. DOI: 10.1107/S1600536810043813/rz2507Isup2.hkl

Additional supplementary materials:  crystallographic information; 3D view; checkCIF report

## Figures and Tables

**Table 1:** Hydrogen-bond geometry (Å, °)

*D*—H⋯*A*	*D*—H	H⋯*A*	*D*⋯*A*	*D*—H⋯*A*
O1*W*—H1*F*⋯O2^i^	0.93	1.84	2.759 (3)	176
O1*W*—H1*E*⋯N3^ii^	0.92	1.96	2.879 (3)	173
O1—H1⋯O1*W*^iii^	0.82	1.75	2.558 (3)	167

Symmetry codes: (i) 
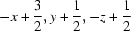
; (ii) 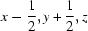; (iii) 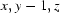.

**Fig. 1.**
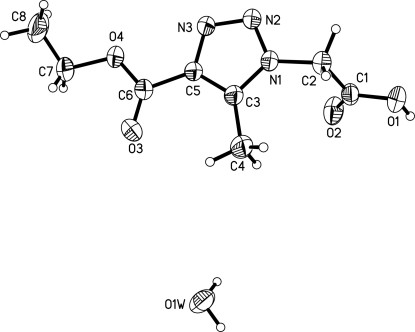
The molecular structure of the title compound, showing the atomic numbering scheme. The displacement ellipsoids are drawn at the 30% probability level.

**Fig. 2.**
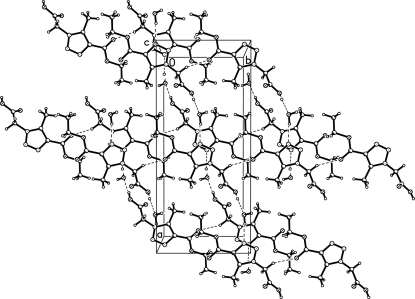
Packing diagram of the title compound, showing the structure along the c axis.

## supplementary crystallographic information

### Comment

Triazole derivatives have attracted considerable attention due to their biological activities (Olesen *et al.*, 2003; Tian *et al.*, 2005). Recently, we have reported the crystal structures of a few triazole compounds (Lin *et al.*, 2008; Xiao *et al.*, 2008; Zhao, 2009). As an extension of our work on the structural characterization of triazole derivatives, the crystal structure of the title compound is reported here.

In the molecule of the title compound (Fig. 1) bond lengths and angles have normal values. The crystal packing is stabilized by strong intermolecular O—H···N and O—H···O hydrogen bonds involving the triazole and water molecules (Fig. 2; Table 1) forming double layers parallel to the *ab* plane.

### Experimental

The title compound was prepared from 2-azidoacetic acid according to the reported method (El Khadem *et al.*, 1968). Colourless prismatic crystal suitable for X-ray analysis were obtained by slow evaporation of a 95% ethanol/water solution.

### Refinement

The water H atoms were located from a difference Fourier map but not refined [*U*_iso_(H) = 1.5*U*_eq_(O)]. All other H atoms were fixed geometrically and treated as riding with C—H = 0.96–0.97 Å, O—H = 0.82 Å, and with *U*_iso_(H) = 1.2*U*_eq_(C) or 1.5*U*_eq_(C, O) for methyl and carboxy H atoms.

### Crystal data

**Table:**

C_8_H_11_N_3_O_4_·H_2_O	*F*(000) = 976
*M**_r_* = 231.21	*D*_x_ = 1.339 Mg m^−^^3^
Monoclinic, *C*2/*c*	Mo *K*α radiation, λ = 0.71073 Å
Hall symbol: -C 2yc	Cell parameters from 2099 reflections
*a* = 18.6082 (15) Å	θ = 2.7–27.5°
*b* = 8.2295 (15) Å	µ = 0.11 mm^−^^1^
*c* = 14.986 (2) Å	*T* = 295 K
β = 92.050 (5)°	Prism, colourless
*V* = 2293.4 (6) Å^3^	0.35 × 0.32 × 0.28 mm
*Z* = 8	

### Data collection

**Table:**

Rigaku SCXmini diffractometer	2490 independent reflections
Radiation source: fine-focus sealed tube	1536 reflections with *I* > 2σ(*I*)
graphite	*R*_int_ = 0.051
Detector resolution: 13.6612 pixels mm^-1^	θ_max_ = 27.0°, θ_min_ = 2.7°
CCD_Profile_fitting scans	*h* = −23→23
Absorption correction: multi-scan (*CrystalClear*; Rigaku, 2005)	*k* = −10→10
*T*_min_ = 0.960, *T*_max_ = 0.970	*l* = −19→19
10958 measured reflections	

### Refinement

**Table:**

Refinement on *F*^2^	Primary atom site location: structure-invariant direct methods
Least-squares matrix: full	Secondary atom site location: difference Fourier map
*R*[*F*^2^ > 2σ(*F*^2^)] = 0.066	Hydrogen site location: inferred from neighbouring sites
*wR*(*F*^2^) = 0.189	H-atom parameters constrained
*S* = 1.09	*w* = 1/[σ^2^(*F*_o_^2^) + (0.0861*P*)^2^ + 0.6922*P*] where *P* = (*F*_o_^2^ + 2*F*_c_^2^)/3
2490 reflections	(Δ/σ)_max_ < 0.001
148 parameters	Δρ_max_ = 0.28 e Å^−^^3^
0 restraints	Δρ_min_ = −0.20 e Å^−^^3^

### Special details

**Table:**

Geometry. All e.s.d.'s (except the e.s.d. in the dihedral angle between two l.s. planes) are estimated using the full covariance matrix. The cell e.s.d.'s are taken into account individually in the estimation of e.s.d.'s in distances, angles and torsion angles; correlations between e.s.d.'s in cell parameters are only used when they are defined by crystal symmetry. An approximate (isotropic) treatment of cell e.s.d.'s is used for estimating e.s.d.'s involving l.s. planes.
Refinement. Refinement of *F*^2^ against ALL reflections. The weighted *R*-factor *wR* and goodness of fit *S* are based on *F*^2^, conventional *R*-factors *R* are based on *F*, with *F* set to zero for negative *F*^2^. The threshold expression of *F*^2^ > σ(*F*^2^) is used only for calculating *R*-factors(gt) *etc*. and is not relevant to the choice of reflections for refinement. *R*-factors based on *F*^2^ are statistically about twice as large as those based on *F*, and *R*- factors based on ALL data will be even larger.

### Fractional atomic coordinates and isotropic or equivalent isotropic displacement parameters (Å^2^)

**Table:**

	*x*	*y*	*z*	*U*_iso_*/*U*_eq_	
C1	0.81442 (14)	−0.2524 (3)	0.37722 (19)	0.0552 (6)	
C2	0.87508 (14)	−0.2098 (3)	0.44180 (18)	0.0611 (7)	
H2A	0.8554	−0.1835	0.4991	0.073*	
H2B	0.9059	−0.3041	0.4502	0.073*	
C3	0.90266 (14)	0.0868 (3)	0.41202 (18)	0.0557 (6)	
C4	0.83230 (16)	0.1554 (4)	0.4359 (3)	0.0936 (12)	
H4A	0.7981	0.1402	0.3871	0.140*	
H4B	0.8376	0.2694	0.4481	0.140*	
H4C	0.8156	0.1012	0.4880	0.140*	
C5	0.96537 (13)	0.1568 (3)	0.38496 (18)	0.0542 (6)	
C6	0.98182 (15)	0.3313 (3)	0.3765 (2)	0.0631 (7)	
N1	0.91816 (11)	−0.0742 (2)	0.41279 (14)	0.0552 (6)	
N2	0.98618 (12)	−0.1025 (3)	0.38793 (17)	0.0658 (6)	
N3	1.01479 (11)	0.0386 (2)	0.37055 (17)	0.0629 (6)	
O1	0.77303 (11)	−0.3646 (2)	0.41090 (13)	0.0687 (6)	
H1	0.7401	−0.3861	0.3750	0.103*	
O2	0.80534 (12)	−0.1933 (3)	0.30435 (15)	0.0828 (7)	
O3	0.93857 (12)	0.4367 (2)	0.38956 (19)	0.0963 (8)	
O4	1.04841 (10)	0.3565 (2)	0.35401 (16)	0.0787 (7)	
O1W	0.66222 (12)	0.5429 (3)	0.31857 (17)	0.1016 (9)	
H1E	0.6157	0.5328	0.3366	0.152*	
H1F	0.6724	0.4673	0.2751	0.152*	
C7	1.07274 (18)	0.5254 (4)	0.3491 (3)	0.0975 (13)	
H7A	1.0559	0.5733	0.2931	0.117*	
H7B	1.0531	0.5877	0.3975	0.117*	
C8	1.1478 (2)	0.5300 (5)	0.3554 (4)	0.1357 (19)	
H8A	1.1645	0.4680	0.4063	0.204*	
H8B	1.1635	0.6406	0.3620	0.204*	
H8C	1.1669	0.4846	0.3022	0.204*	

### Atomic displacement parameters (Å^2^)

**Table:**

	*U*^11^	*U*^22^	*U*^33^	*U*^12^	*U*^13^	*U*^23^
C1	0.0569 (15)	0.0444 (13)	0.0649 (16)	−0.0084 (11)	0.0111 (12)	−0.0044 (12)
C2	0.0590 (16)	0.0525 (15)	0.0720 (17)	−0.0113 (12)	0.0030 (13)	0.0027 (13)
C3	0.0483 (14)	0.0482 (14)	0.0705 (16)	−0.0026 (11)	0.0034 (12)	−0.0085 (12)
C4	0.0614 (19)	0.068 (2)	0.154 (3)	−0.0031 (16)	0.031 (2)	−0.017 (2)
C5	0.0462 (14)	0.0448 (13)	0.0713 (16)	−0.0019 (11)	−0.0012 (12)	−0.0070 (12)
C6	0.0556 (16)	0.0482 (14)	0.085 (2)	−0.0050 (13)	−0.0067 (14)	−0.0044 (13)
N1	0.0482 (12)	0.0445 (11)	0.0729 (14)	−0.0073 (9)	0.0042 (10)	−0.0021 (10)
N2	0.0493 (13)	0.0470 (12)	0.1013 (18)	−0.0040 (10)	0.0066 (12)	0.0008 (12)
N3	0.0462 (12)	0.0470 (12)	0.0956 (17)	−0.0039 (9)	0.0059 (11)	−0.0033 (11)
O1	0.0652 (12)	0.0675 (12)	0.0739 (12)	−0.0251 (10)	0.0078 (9)	−0.0006 (10)
O2	0.0906 (16)	0.0848 (15)	0.0724 (13)	−0.0321 (12)	−0.0074 (11)	0.0157 (12)
O3	0.0744 (15)	0.0479 (11)	0.167 (2)	0.0032 (11)	0.0108 (15)	−0.0080 (13)
O4	0.0532 (11)	0.0499 (11)	0.1330 (19)	−0.0134 (9)	0.0037 (11)	0.0032 (11)
O1W	0.0557 (13)	0.122 (2)	0.128 (2)	−0.0154 (12)	0.0168 (13)	−0.0639 (16)
C7	0.075 (2)	0.0488 (17)	0.168 (4)	−0.0179 (15)	−0.011 (2)	0.014 (2)
C8	0.095 (3)	0.094 (3)	0.216 (5)	−0.049 (2)	−0.031 (3)	0.050 (3)

### Geometric parameters (Å, °)

**Table:**

C1—O2	1.202 (3)	C6—O3	1.204 (3)
C1—O1	1.315 (3)	C6—O4	1.312 (3)
C1—C2	1.502 (4)	N1—N2	1.352 (3)
C2—N1	1.450 (3)	N2—N3	1.307 (3)
C2—H2A	0.9700	O1—H1	0.8200
C2—H2B	0.9700	O4—C7	1.465 (3)
C3—N1	1.356 (3)	O1W—H1E	0.9194
C3—C5	1.375 (3)	O1W—H1F	0.9251
C3—C4	1.481 (4)	C7—C8	1.396 (5)
C4—H4A	0.9600	C7—H7A	0.9700
C4—H4B	0.9600	C7—H7B	0.9700
C4—H4C	0.9600	C8—H8A	0.9600
C5—N3	1.361 (3)	C8—H8B	0.9600
C5—C6	1.475 (3)	C8—H8C	0.9600
			
O2—C1—O1	124.6 (3)	O3—C6—C5	123.0 (3)
O2—C1—C2	124.7 (2)	O4—C6—C5	112.1 (2)
O1—C1—C2	110.7 (2)	N2—N1—C3	111.5 (2)
N1—C2—C1	113.4 (2)	N2—N1—C2	118.9 (2)
N1—C2—H2A	108.9	C3—N1—C2	129.4 (2)
C1—C2—H2A	108.9	N3—N2—N1	107.1 (2)
N1—C2—H2B	108.9	N2—N3—C5	108.7 (2)
C1—C2—H2B	108.9	C1—O1—H1	109.5
H2A—C2—H2B	107.7	C6—O4—C7	117.3 (2)
N1—C3—C5	103.2 (2)	H1E—O1W—H1F	111.4
N1—C3—C4	124.1 (2)	C8—C7—O4	109.4 (3)
C5—C3—C4	132.7 (2)	C8—C7—H7A	109.8
C3—C4—H4A	109.5	O4—C7—H7A	109.8
C3—C4—H4B	109.5	C8—C7—H7B	109.8
H4A—C4—H4B	109.5	O4—C7—H7B	109.8
C3—C4—H4C	109.5	H7A—C7—H7B	108.2
H4A—C4—H4C	109.5	C7—C8—H8A	109.5
H4B—C4—H4C	109.5	C7—C8—H8B	109.5
N3—C5—C3	109.4 (2)	H8A—C8—H8B	109.5
N3—C5—C6	122.6 (2)	C7—C8—H8C	109.5
C3—C5—C6	127.9 (2)	H8A—C8—H8C	109.5
O3—C6—O4	124.8 (3)	H8B—C8—H8C	109.5
			
O2—C1—C2—N1	7.1 (4)	C5—C3—N1—C2	175.1 (3)
O1—C1—C2—N1	−173.6 (2)	C4—C3—N1—C2	−5.1 (4)
N1—C3—C5—N3	0.2 (3)	C1—C2—N1—N2	−110.4 (3)
C4—C3—C5—N3	−179.6 (3)	C1—C2—N1—C3	74.9 (3)
N1—C3—C5—C6	−176.4 (3)	C3—N1—N2—N3	−0.4 (3)
C4—C3—C5—C6	3.8 (5)	C2—N1—N2—N3	−176.0 (2)
N3—C5—C6—O3	−179.6 (3)	N1—N2—N3—C5	0.5 (3)
C3—C5—C6—O3	−3.4 (5)	C3—C5—N3—N2	−0.4 (3)
N3—C5—C6—O4	−0.1 (4)	C6—C5—N3—N2	176.4 (3)
C3—C5—C6—O4	176.1 (3)	O3—C6—O4—C7	3.4 (5)
C5—C3—N1—N2	0.1 (3)	C5—C6—O4—C7	−176.1 (3)
C4—C3—N1—N2	179.9 (3)	C6—O4—C7—C8	159.6 (4)

### Hydrogen-bond geometry (Å, °)

**Table:**

*D*—H···*A*	*D*—H	H···*A*	*D*···*A*	*D*—H···*A*
O1W—H1F···O2^i^	0.93	1.84	2.759 (3)	176
O1W—H1E···N3^ii^	0.92	1.96	2.879 (3)	173
O1—H1···O1W^iii^	0.82	1.75	2.558 (3)	167

Symmetry codes: (i) −*x*+3/2, *y*+1/2, −*z*+1/2; (ii) *x*−1/2, *y*+1/2, *z*; (iii) *x*, *y*−1, *z*.
